# Location of Symbionts in the Whitefly *Bemisia tabaci* Affects Their Densities during Host Development and Environmental Stress

**DOI:** 10.1371/journal.pone.0091802

**Published:** 2014-03-14

**Authors:** Qi Su, Wen Xie, Shaoli Wang, Qingjun Wu, Murad Ghanim, Youjun Zhang

**Affiliations:** 1 College of Plant Protection, Hunan Agricultural University, Changsha, Hunan, People’s Republic of China; 2 Department of Plant Protection, Institute of Vegetables and Flowers, Chinese Academy of Agricultural Sciences, Beijing, People’s Republic of China; 3 Department of Entomology, The Agricultural Research Organization (ARO), Volcani Center, Bet Dagan, Israel; University of Minnesota, United States of America

## Abstract

Bacterial symbionts often enhance the physiological capabilities of their arthropod hosts and enable their hosts to expand into formerly unavailable niches, thus leading to biological diversification. Many arthropods, including the worldwide invasive whitefly *Bemisia tabaci*, have individuals simultaneously infected with symbionts of multiple genera that occur in different locations in the host. This study examined the population dynamics of symbionts that are located in different areas within *B. tabaci*. While densities of *Portiera* and *Hamiltonella* (which are located in bacteriocytes) appeared to be well-regulated during host development, densities of *Rickettsia* (which are not located in bacteriocytes) were highly variable among individual hosts during host development. Host mating did not significantly affect symbiont densities. Infection by *Tomato yellow leaf curl virus* did not affect *Portiera* and *Hamiltonella* densities in either sex, but increased *Rickettsia* densities in females. High and low temperatures did not affect *Portiera* and *Hamiltonella* densities, but low temperature (15°C) significantly suppressed *Rickettsia* densities whereas high temperature (35°C) had little effect on *Rickettsia* densities. The results are consistent with the view that the population dynamics of bacterial symbionts in *B. tabaci* are regulated by symbiont location within the host and that the regulation reflects adaptation between the bacteria and insect.

## Introduction

Bacteria and insects commonly form intimate, symbiotic associations that result from co-evolution. Many of these symbioses are ancient and highly specialized, and these include the obligatory, primary symbioses between *Buchnera aphidicola* and aphids, *Carsonella ruddii* and psyllids, *Tremblaya princeps* and mealybugs, and *Portiera aleyrodidarum* and whiteflies [1]. By synthesizing essential amino acids, these primary symbiotic bacteria provide essential nutrients to insects whose diets (e.g., phloem sap) are typically nutrient-poor [1], [2]. The primary bacterial symbionts are housed within specialized cells called bacteriocytes that aggregate to form an organ termed the bacteriome; bacteriocytes and the primary symbionts within are transovarially transmitted from mothers to offspring [1], [3].

In contrast to primary symbionts, secondary symbionts may not be required for host survival but may play important roles in protection from natural enemies and pathogens [4]–[6], adaptation to a wide range of food plants [7], genetic differentiation [8], and reproduction [9]. The localizations of secondary symbionts in their hosts are diverse and vary in the host body. For example, secondary symbiots have been detected in insect tissues such as the primary and secondary bacteriocytes [10]–[12], Malpighian tubules [13], hemolymph [14], [15], reproductive organs [16], and salivary glands [17]. Like primary symbionts, intracellular secondary symbionts are generally vertically transmitted; in addition, some strains of symbionts also located outside the bacteriocytes and undergo occasional horizontal transfer [18], [19].

The sweet potato whitefly, *Bemisia tabaci* (Gennadius) (Hemiptera: Aleyrodidae), has been regarded as a species complex consisting of many putative species that differ genetically and biologically [20], [21]. The most widespread and damaging groups are B and Q [recently termed Middle East Asia Minor 1 and Mediterranean, respectively] [20], both of which have caused considerable losses to agricultural crops as a consequence of their feeding on phloem sap and transmission of plant viruses, especially begomoviruses. The relationships between begomoviruses and whiteflies are complex. For example, *Tomato yellow leaf curl virus* (TYLCV) is transmitted by *B. tabaci* in a persistent, circulative, non-propagative manner [22], [23], and *B. tabaci* B can transmit TYLCV DNA horizontally during sexual transmission and vertically via transovarial passage, albeit with low frequency [24]. This suggests that TYLCV invades the reproductive system of the viruliferous whiteflies. In addition, when *B. tabaci* B viruliferous whiteflies were allowed to feed on cotton, a non-host plant of the virus, the presence of TYLCV within the whitefly reduced whitefly survival by approximately 11% and fecundity by approximately 21% [25]. Collectively, these results indicate that TYLCV may directly affect whiteflies by inducing changes in whitefly physiology and immune responses.

Previous studies have reported that in addition to the primary symbiont *Portiera*, two secondary symbionts, *Hamiltonella* and *Rickettsia*, occur in *B. tabaci* B populations in China [26], [27] and Israel [11], [28]. These three symbionts differ in co-evolutionary histories with their hosts, localization patterns in their hosts, and transmission modes. The obligate mutualist *Portiera* (Gammaproteobacteria: Halomonadaceae) represents an ancient and highly specialized association, is found only in bacteriocytes, and exhibits a pattern of strict co-speciation with its whitefly host species [29]. In contrast, *Hamiltonella* (Enterobacteriaceae) does not display a concordant evolutionary history with the whitefly host [30], which suggests a more recent association, but is also restricted to bacteriocytes that normally house *Portiera* [11], [12]. The role of *Hamiltonella* in whiteflies is poorly understood, although it has been associated with transmission of plant viruses by *B. tabaci* [31], and its specific elimination has been reported to result in less efficient virus transmission [32]. Like *Hamiltonella*, *Rickettsia* (Alphaproteobacteria: Rickettsiaceae) exhibits a relatively recent association with its hosts but a more variable tissue tropism, inhabiting bacteriocytes and a variety of other somatic tissues [11], [33]. A wide range of phenotypic effects on the host, such as enhanced heat tolerance [34], increased fitness [35], and increased susceptibility to insecticides [36], have been ascribed to *Rickettsia* infections.

**Figure 1:**
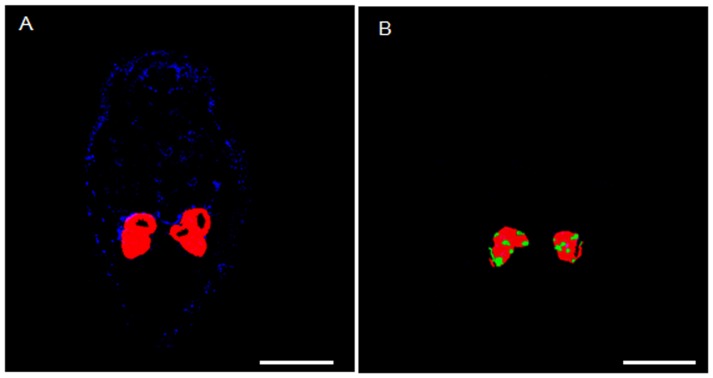
Whole-mount FISH analysis of the *B. tabaci* B nymphs used in this study. (A) Overlay of *Portiera* and *Rickettsia* on dark field. (B) Overlay of *Portiera* and *Hamiltonella* on dark field. The epifluorescent images were generated artificially by combining two relevant monochrome images obtained with the same exposure time in the microscope. Red: *Portiera*; Blue: *Rickettsia*. Green: *Hamiltonella*. Bars = 100 µm.

The localization of a symbiont in its host could affect its horizontal transmission from whitefly to parasitoid wasp [18], symbiont dynamics in the whitefly [37], and whitefly response to external stresses, such as parasitization [38]. In the current study of symbiont location in the host, we took advantage of the fact that the individuals in our glasshouse population of *B. tabaci* B are simultaneously infected with multiple symbiont genera that differ in location (Fig. 1). We used this *B. tabaci* B population to examine the population dynamics of multiple microbial symbionts during development in the same host individuals. We also investigated the influence of extrinsic factors that could impact symbiotic homeostasis, such as viral infection and temperature. We predicted that bacterial symbionts of *B. tabaci* B would exhibit population dynamics that were coordinated with host development and critical life events.

## Materials and Methods

### Whitefly Rearing

The *B. tabaci* B population used in this study was originally collected from cabbage, *Brassica oleracea* var. Jingfeng1, in 2004, in the Haidian District of Beijing, China. It was subsequently maintained on cotton, *Gossypium herbaceum* L., cv DP99B, in separate screen cages under natural lighting and ambient temperature (26±2°C) in a glasshouse. The purity of the population was monitored by sampling 20 adults every generation based on the CAPS (cleavage amplified polymorphic sequence) of *mitochondrial cytochrome oxidase I* (*mtCOI*) with the restriction endonucleases *Vsp*I [39]. At the time of this study, this host-adapted population had been cultured for 9 years (approximately 135 generations) under the same conditions without exposure to any insecticides. Periodic diagnostic screening revealed that this population contained *Hamiltonella* and *Rickettsia* (which always occur together in *B. tabaci* B individuals in China), and *Portiera* (the primary symbiont of whiteflies), but did not contain *Wolbachia*, *Arsenophonus*, or *Fritschea*. This occurrence of bacterial symbionts in our *B. tabaci* B population is consistent with the results presented by Chu et al. (2011) [26] and Pan et al. (2012b) [27] for field populations from China. *Rickettsia* in these whiteflies was distributed throughout the body cavity excluding the bacteriocytes (Fig. 1A), while *Portiera* and *Hamiltonella* were located in the bacteriocytes and were never detected in any other host organ by fluorescence *in situ* hybridization (FISH) analysis (Fig. 1B). The failure to detect *Portiera* and *Hamiltonella* in other host organs is consistent with previous publications [11], [40].

### Sample Collection and DNA Extraction

Total DNA was extracted using the Chelex DNA extraction protocol [41] from nymphs, teneral adults (newly emerged adults; unfed), and 1- and 2-week-old virgin adults on cotton plants. DNA was similarly extracted from 1- to 4-week-old mated females. The nymphs were recognized as one of four instars (L1, L2, L3, and L4) based on morphological characteristics [42]. *Portiera* and *Hamiltonella* were quantified for all of these host stages. For *Rickettsia* quantification, however, our preliminary experiment revealed no significant differences in *Rickettsia* densities during nymph, teneral, and adult development in either sex or among individuals of the same age class. Therefore, we separated host development into three categories: nymph, teneral, and adult. In this case, the variability was too large to allow for mean determination. Although sample sizes for *Rickettsia* density estimates were increased due to the high variability, this did not result in conformity. Hence, the median rather than the mean was used as a measure of *Rickettsia* density during host development. For the offspring from individual mothers, the mated female whiteflies (2 days after emergence) were transferred individually to clip-cages attached to leaves (the third leaf from the top) of cotton plants in a cage. The females in the clip-cages were transferred to a new plant every day, and they were removed from the last plant after 4 days. The four groups of eggs were kept separate, and the adults were collected when they emerged. The collected insects were stored at −20°C, processed for DNA isolation, and assayed for symbiont density by q-PCR as described later.

### Effect of *B. tabaci* Virus Status on Endosymbiont Population Dynamics

Because *B. tabaci* is an important vector of TYLCV and the impact of a plant virus on whitefly–endosymbiont interactions has not been previously reported, we evaluated how TYLCV infection affected endosymbionts in *B. tabaci* B. Symbiont densities were examined following host acquisition of TYLCV. TYLCV-infected tomato plants were obtained by agro-inoculation as previously described [43]. The control tomato plants were mock inoculated using the *Agrobacterium tumefaciens* strain EHA105 empty vector. Virus infection of test plants was assessed based on symptoms and was confirmed by PCR using a previously described procedure [44]. To obtain non-viruliferous and viruliferous whiteflies, newly emerged, non-viruliferous *B. tabaci* adults were released onto two TYLCV-infected tomato plants in one cage and two healthy tomato plants in another cage. After a 48-h acquisition access period (AAP), the non-viruliferous and viruliferous whiteflies were subsequently maintained on cotton plants, a non-host of TYLCV. PCR analyses showed that viral DNA was present in 100% of the whiteflies after a 48-h AAP on virus-infected tomato plants and that 90% of the whiteflies still carried virus after 2 weeks of feeding on cotton plants (data not shown). Genomic copy numbers of all three symbionts were estimated for whiteflies using q-PCR at 1- and 2-weeks following viral challenge. This experiment was conducted in climate chambers at 27±2°C with 14 h light/10 h darkness and 70%±10% relative humidity.

### Temperature Treatments

Twenty pairs of newly-emerged whiteflies were released onto 5 cotton plants (four pairs per plant). After a 3-day oviposition period at 25±1°C, the adults were removed. The cotton plants with eggs were then maintained at 15, 25, or 35°C in climatic incubators (precision ±0.2°C; MHT350; Sanyo Electric Co., Ltd, Osaka, Japan). Adult insects were collected from each treatment 1 to 2 days after the onset of adult emergence. From the collected whiteflies, 10 pairs were used to initiate the next generation (they were added to 5 replicate plants, with 2 pairs per plant) and the remaining whiteflies were preserved in 80% ethanol for subsequent molecular analyses. The intervals between the start of oviposition on the cotton leaves and the collection of adult whiteflies of the next generation were 63 to 66 days at 15°C, 18 to 21 days at 25°C, and 14 to 16 days at 35°C. In this way, the insects were maintained at different temperatures through three successive generations. The whiteflies that died during the experiment were discarded. Each temperature treatment for each generation was represented by three replicate plants.

### Quantitative PCR

Bacterial density was quantified by the SYBR Green ROX mix and ABI Prism 7500 Sequence Detection System (Applied Biosystems). *Portiera* was quantified in terms of *16S rRNA* gene copies using primers Port-F (5- TAGTCCACGCTGTAAACG-3) and Port-R (5- AGGCACCCTTCCATCT-3). *Hamiltonella* was quantified in terms of *16S rRNA* gene copies using primers Ham-F (5- GCATCGAGTGAGCACAGTT-3) and Ham-R (5-TATCCTCTCAGACCCGCTAA-3) [32]. *Rickettsia* was quantified in terms of the *gltA* gene using primers glt375-F (5- TGGTATTGCATCG CTTTGGG-3) and glt574-R (5- TTTCTTTAAGCACTGCAGCACG-3) [37]. The *B. tabaci β*-actin gene with primers *β-actin* F (5- TCTTCCAGCCATCCTTCTTG-3) and *β-actin* R (5- CGGTGATTTCCTTCTGCATT-3) [45] was used as an internal standard for data normalization.

Quantitative PCR reactions were carried out in 15-µl volumes containing 6.75 µl of 2.5×Real Master Mix (SYBR Green) (TIANGEN Biotech, Beijing, China), 6.25 µl of RNase-free water, 0.5 µl of forward and reverse primer solution (10 µM each), and 2 µl of DNA. The cycling conditions for symbionts were: 5 min activation at 95°C followed by 40 cycles of 10 s at 95°C, 30 s at 60°C, and 60 s at 72°C. Standard curves were drawn using standard plasmid samples for each symbiont’s gene at concentrations of 10^3^, 10^4^, 10^5^, 10^6^, 10^7^, and 10^8^ copies/µl, and efficiencies for all quantification reactions were >95%. Sterile water was used as the template in the control. Symbiont density is defined as the bacterial gene copy number divided by the host nuclear gene copy number (relative density). DNA from each experimental sample was analyzed to quantify the density of the three symbionts. The real-time quantitative PCR data were quantified with the ABI Prism 7500 Sequence Detection System and accompanying software. All assays were carried out in triplicate in each of 20 biologically independent experiments.

### Fluorescence *in situ* Hybridization (FISH)

FISH analysis of *B. tabaci* nymphs was performed as previously described by Gottlieb et al. (2006) [40] with the probe BTP1-Cy3 (5′-Cy3-TGTCAGTGTCAGCCCAGAAG-3′) to detect *Portiera*, the probe BTH-Cy5 (5′- CCAGATTCCCAGACTTTACTCA-3′) to detect *Hamiltonella*, and the probe Rb1- Cy5 (5′-Cy5-TCCACGTCGCCGTCTTGC-3′) to detect *Rickettsia* [11]. Stained samples were mounted whole and were photographed on a single focal plane viewed with a laser confocal microscope (LSM 510 META, Carl Zeiss). In each figure, we used both probes (Cy3 and Cy5) in the same hybridization performed on a single histological section and we examined the relevant monochrome immediately after we added the probes. For each treatment, at least 50 specimens were examined to confirm reproducibility. Optical sections, 0.7–1.0 µm thick, were made of each specimen. Specificity of detection was confirmed by using the following controls: a no-probe control, an RNase-digested control, and *Rickettsia*-free (samples of the *B. tabaci* Q population [32]) whitefly controls.

### Statistical Analysis

Data were analyzed with SPSS 17.0 (SPSS Inc., Chicago, IL, USA). Paired *t*-tests and multi-way analyses of variance (ANOVAs) plus *post hoc* pairwise comparison of the means were performed to determine whether densities were significantly affected by host development, host sex, virus infection, or temperature treatments. If densities appeared skewed, the data were log transformed to satisfy normality. *F*-tests were applied to assess homogeneity of variances. When necessary, final ANOVA models were modified to account for unequal variances across groups.

## Results

### Population Dynamics of Symbionts during Host Development

**Figure 2:**
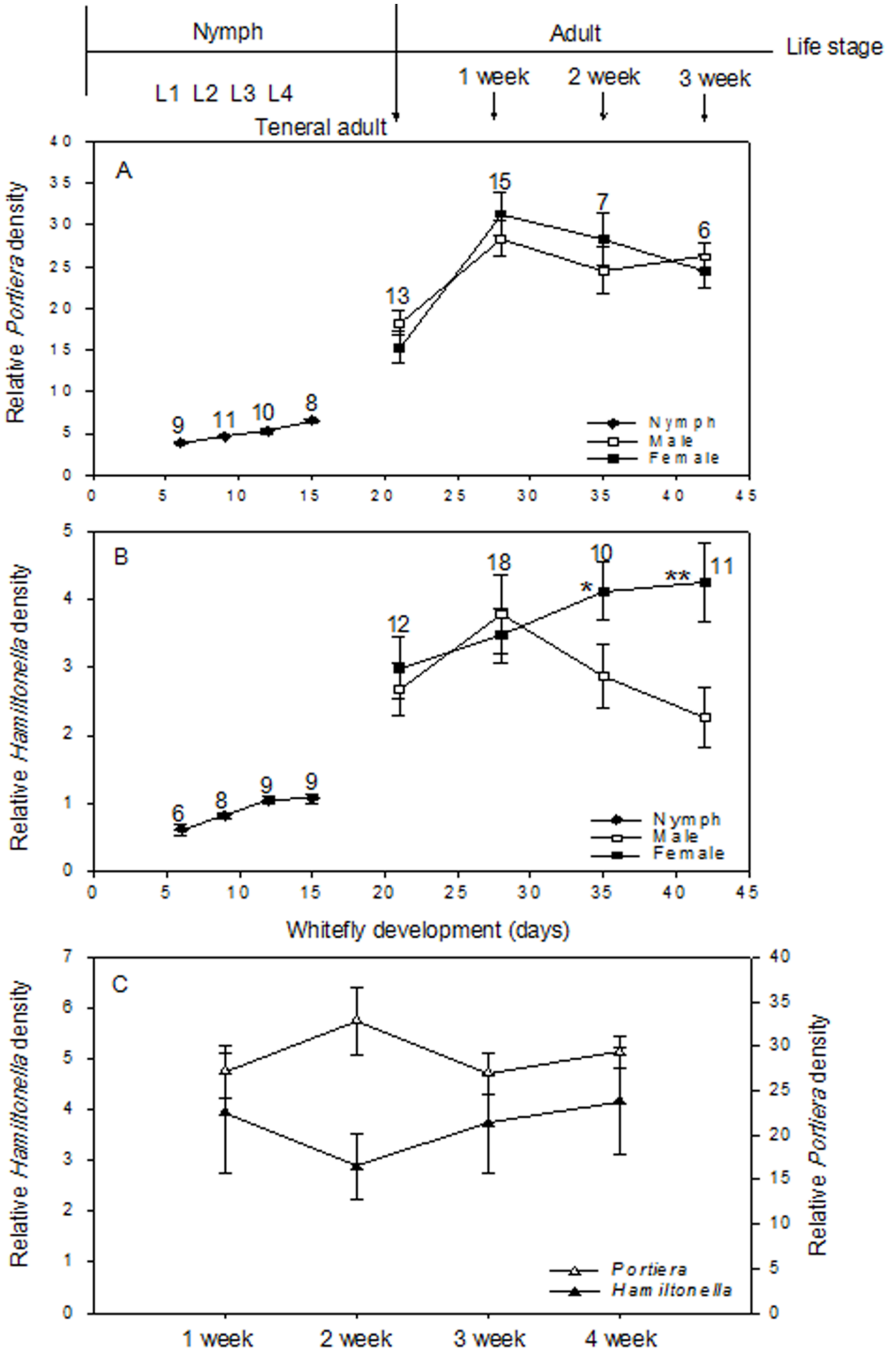
Population density of bacterial symbionts (as indicated by relative copy number of specific genes) during *B. tabaci* B development and as affected by *B. tabaci* B mating status. (A) *Portiera* density. (B) *Hamiltonella* density. Whitefly sexes are indistinguishable before the adult phase (earlier than day 20 after hatch). Sample sizes are indicated above the data symbols for each time point. *, **indicate significant differences between densities in virgin males and females at the indicated time point (*p*<0.01, 0.001; respectively). (C) *Portiera* and *Hamiltonella* densities in 1-, 2-, 3-, and 4-week-old mated females. Values are means±SEM (n ≥10).

**Figure 3:**
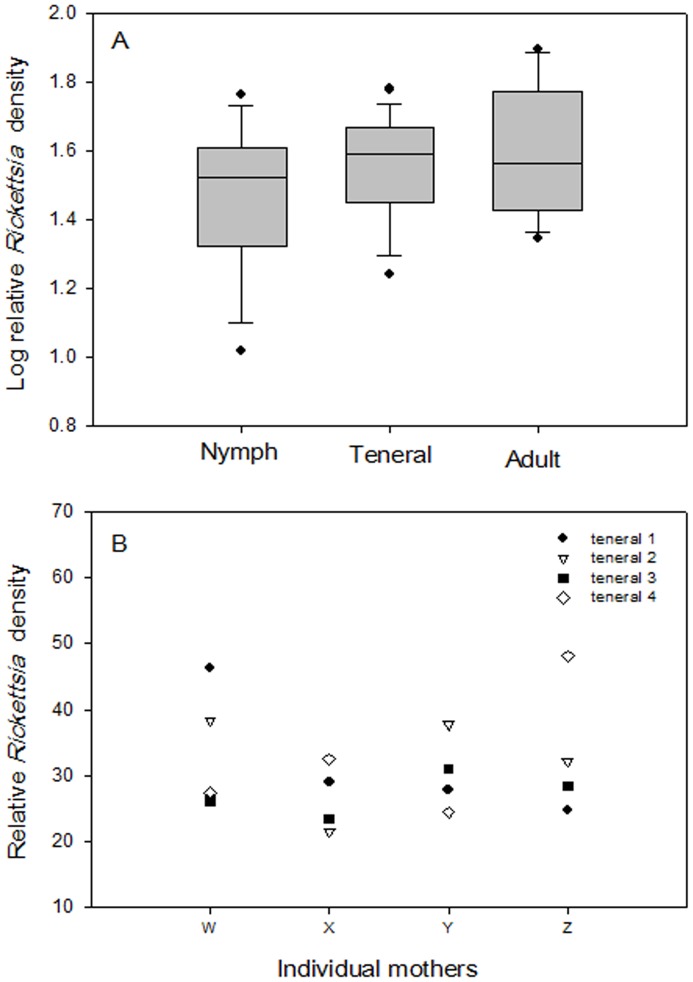
*Rickettsia* density (as indicated by relative copy number of specific genes) during *B. tabaci* B development and in different offspring. (A) Box-and-whisker plots of *Rickettsia* density and distribution of variance during host development. Host development was separated into three stages: nymph (n = 20), teneral (n = 18), and adult (n = 25). The median is indicated by a solid line, the 25th to 75th percentile is boxed, and the 10th and 90th percentiles are indicated by whiskers. (B) *Rickettsia* densities in four offspring (tenerals) of each of four randomly selected mothers (W–Z) determined immediately following eclosion; the offspring from each mother are numbered 1–4 in accordance with their order of deposition.

All three symbionts (*Portiera*, *Hamiltonella*, and *Rickettsia*) were found in all whiteflies. Estimates of symbiont abundance indicated that *Portiera* was maintained at a density one order of magnitude higher than that of *Hamiltonella* in all host developmental stages (Fig. 2A). Both *Portiera* and *Hamiltonella* proliferated in parallel with host cell replication during the immature stages of the hosts (i.e., <15 days) but then significantly increased during the transition from the pupal to the teneral stage (Fig. 2A and 2B). *Portiera* densities were 2.05-fold and 1.55-fold greater in female and male adults in the post-teneral stage than in tenerals (Fig. 2A). Although host sex did not appear to influence *Portiera* density, *Hamiltonella* densities differed in male and virgin female adults (Fig. 2B). *Hamiltonella* densities peaked in 1-week-old adult males and then declined but continued to increase in virgin female adults (Fig. 2B). Because the sex of whiteflies is indistinguishable in nymphs, only data from adult whiteflies were analyzed. *Hamiltonella* densities were significantly affected by the age of *B. tabaci* (*F*_7, 94_ = 4.52, *p = *0.018), the sex of *B. tabaci* (*F*_7, 94_ = 24.26, *p*<0.0001), and by the interaction between these two factors (*F*_7, 94_ = 9.29, *p = *0.001). The median *Rickettsia* densities did not significantly differ among nymph, teneral adult, and adult stages (*F*_2, 60_ = 1.658, *p* = 0.203, Fig. 3A). Furthermore, *Rickettsia* densities of the offspring from each of four mothers varied greatly, i.e., offspring from a particular mother often differed greatly in their *Rickettsia* densities, and this variation was independent of the order of egg deposition (Fig. 3B).

### Effect of Host Mating on Symbiont Density

Symbiont densities from 1-, 2-, 3-, and 4-week-old mated females were quantified to determine whether host reproductive status influences symbiont population dynamics. *Portiera* densities in mated females were similar to those in virgin females (29.04±2.72 bacteria per mated female and 28.07±2.59 per virgin; *t*_1, 28_ = 1.57, *p = *0.519; Fig. 2C vs. 2A). Although *Hamiltonella* densities were similar in 1-week-old mated and virgin females (3.47±0.40 per mated female and 3.94±0.64 per virgin; *t*_1, 30_ = 1.342, *p = *0.692; Fig. 2C vs. 2B), the numbers declined in 2-week-old mated females and then recovered by week 4. *Rickettsia* densities were also not significantly affected by mating (data not shown).

### Effect of TYLCV Infection on Symbiont Density

**Figure 4:**
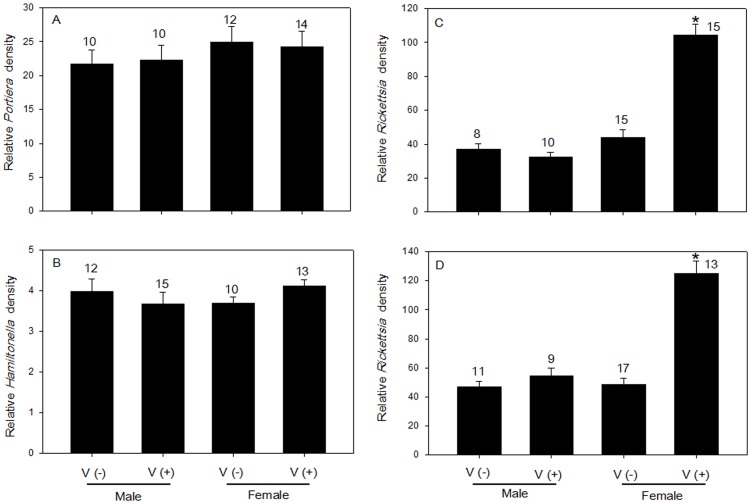
Effect of *Tomato yellow leaf curl virus* infection on symbiont densities (as indicated by relative copy number of specific genes) in *B. tabaci* B. (A) *Portiera* density in 2-week-old males and females. (B) *Hamiltonella* density in 2-week-old males and females. (C) *Rickettsia* density in 2-week-old males and females. (D) *Rickettsia* density in 4-week-old males and females; V(–) (non-viruliferous), V(+) (viruliferous). Sample sizes are indicated above the bars. *indicates significant a significant (*p*<0.001) difference between that treatment and the other treatments. Values are means±SE.

**Figure 5:**
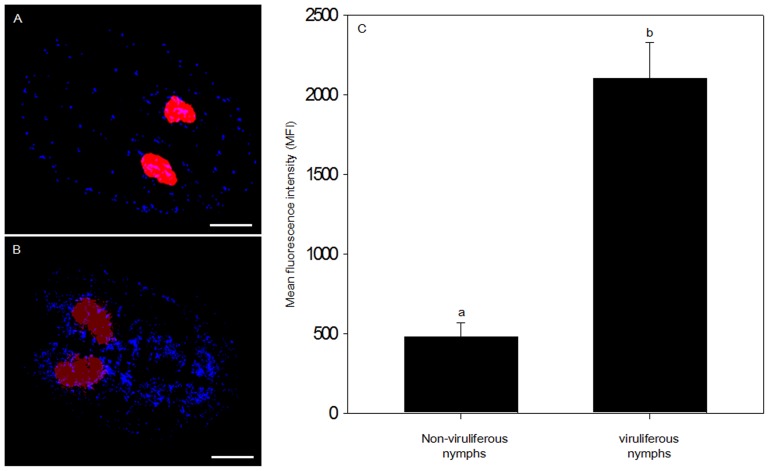
Fluorescence *in situ* hybridization of *B. tabaci* nymphs exposed to TYLCV. (A) Double FISH analysis of a non-viruliferous *B. tabaci* nymph with specific probes for *Portiera* (red) and *Rickettsia* (blue). (B) Double FISH analysis of a viruliferous *B. tabaci* nymph with specific probes for *Portiera* (red) and *Rickettsia* (blue). Bars = 100 µm. (C) Mean fluorescence intensity (MFI). Fluorescence was quantified from 20 viruliferous nymphs and 20 non-viruliferous nymphs by counting the pixels in the corresponding image fields in each group. Pixel intensity was then quantified and finally expressed as MFI. Different letters indicate significant differences between virus treatments (*P*<0.05).

*Portiera* densities did not differ in 2-week-old viruliferous and non-viruliferous whiteflies (*F*_3, 42_ = 2.679, *p* = 0.111; Fig. 4A); similar results were obtained 1 week after viral challenge (data not shown). *Hamiltonella* densities in these same individuals were also unaffected by virus status of the host (at 2 weeks, *F*_3, 46_ = 1.869, *p* = 0.26; Fig. 4B). *Rickettsia* densities in 2- and 4-week-old whiteflies were significantly affected by virus infection status (2 week: *F*_3, 44_ = 33.57, *p*<0.0001; 4 week: *F*_3, 44_ = 42.51, *p*<0.0001), by *B. tabaci* sex (*F*_3, 44_ = 51.38 and *p*<0.0001 at 2 weeks; *F*_3, 44_ = 38.84 and *p*<0.0001 at 4 weeks), and by the interaction between these two factors (*F*_3, 44_ = 45.89 and *p*<0.0001 at 2 weeks; *F*_3, 44_ = 35.73 and *p*<0.0001 at 4 weeks). *Rickettsia* densities did not differ in 2-week-old viruliferous and non-viruliferous males but were much greater in 2-week-old viruliferous than non-viruliferous females (*F*_1, 28_ = 75.78, *p*<0.0001, Fig. 4C) and in 4-week-old viruliferous than non-viruliferous females (*F*_1, 28_ = 72.89, *p*<0.0001, Fig. 4D), suggesting either a sex-specific opportunist role for this symbiont or loss of host control in females upon virus challenge. The increase in *Rickettsia* densities in viruliferous nymphs was also confirmed by FISH analysis (Fig. 5A–C).

### Effect of Temperature on Symbiont Density

**Figure 6:**
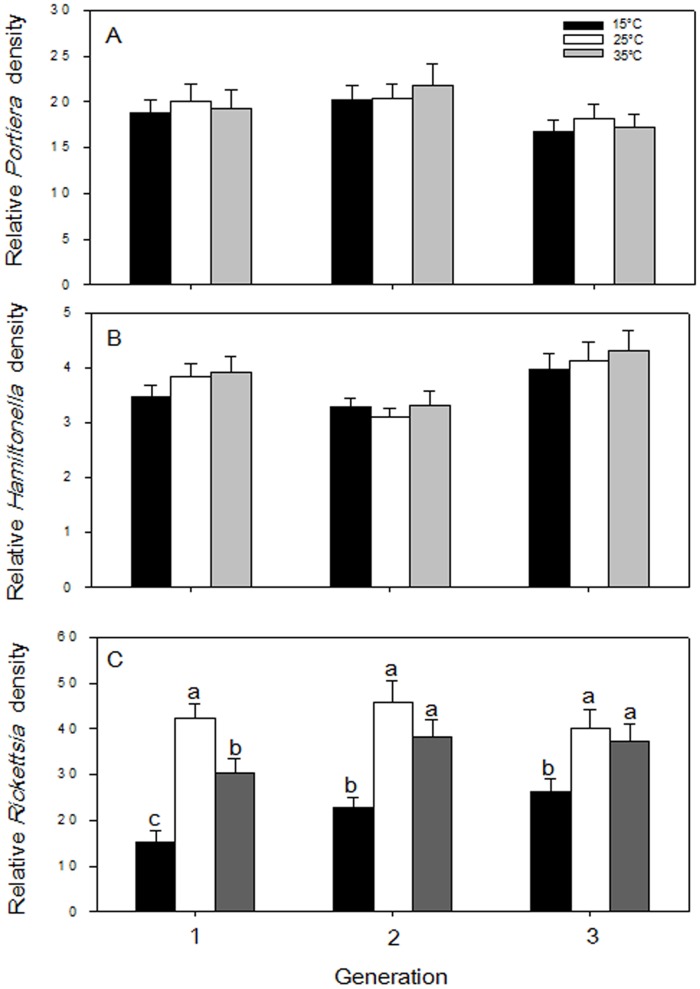
Symbiont density (as indicated by relative copy number of specific genes) in *B. tabaci* whiteflies through three successive host generations maintained under different temperature conditions. (A) *Portiera* density. (B) *Hamiltonella* density. (C) *Rickettsia* density. Values are means±SE (n ≥10). Different letters indicate significant differences among temperature treatments in the same generation (*P*<0.05).

Three successive generations of whiteflies were subjected to temperature treatments, and the symbionts were quantified in adults after each generation. *Portiera* and *Hamiltonella* densities were unaffected by temperature or generation (Fig. 6A and 6B). *Rickettsia* densities were significantly affected by the temperature (*F*_8, 94_ = 33.87, *p*<0.0001) but not by the generation (*F*_8, 94_ = 3.33, *p = *0.059) or the interaction between these two factors (*F*_8, 94_ = 1.21, *p = *0.342). However, *Rickettsia* densities at 35°C were significantly lower than those at 25°C in the first generation (ANOVA, *p = *0.019) but recovered in the subsequent two generations (Fig. 6C). In all three generations, *Rickettsia* densities were significantly lower at 15°C than at 25°C (ANOVA, 1^st^: *p = *0.006; 2^nd^: *p = *0.017; 3^rd^: *p = *0.037) (Fig. 6C).

## Discussion

Our findings demonstrate that TYLCV infection and low temperatures affect *Rickettsia* densities but not *Portiera* or *Hamiltonella* densities in *B. tabaci* B. That the responses to virus infection and temperature differed between these symbionts probably reflects the length of the association between whitefly and symbiont, the functions of the symbionts, and the level of symbiont integration with host biology.

### Population Dynamics of Symbionts during Host Development

The low variability in *Portiera* and *Hamiltonella* densities among immature whitefly individuals of similar ages suggests an adaptive regulation during host development (Fig. 2A and 2B). After remaining at low levels during the development of immature host stages, the densities of both *Portiera* and *Hamiltonella* increased substantially in the teneral adult stage (24 h after eclosion). This increase in the density of both symbionts may be either a host- or symbiont-mediated response resulting from the transition to a life stage independent of the maternal environment. Although the median *Rickettsia* densities were similar throughout whitefly development (Fig. 3A), the densities were highly variable among different whitefly individuals of similar ages (Fig. 3B). The potential cause of the variability in *Rickettsia* densities could be the unequal vertical transmission into progeny or stochastic variation in *Rickettsia* replication rates because we detected high variability among the progeny from the same mother. A previous study found that *Rickettsia* infection conferred a reproductive advantage (female bias) to the whiteflies [35], and hosts may be expected to exhibit a relaxed density control over these infections. Our results for *Rickettsia* are also similar to those reported for *Wolbachia*, a parasitic bacterium whose densities vary greatly among individual hosts of similar ages [46], [47]. It will now be interesting to determine whether the variations in *Rickettsia* densities observed in whitefly individuals of similar age leads to differences in the intensity of the potential physiological modifications that the bacterium causes, such as male mortality or parthenogenesis, as has been reported for *Rickettsia* in other host systems [48]–[50].

### *Hamiltonella* May Provide Benefits to the Whitefly

The densities of *Hamiltonella* (but not of *Portiera*) were substantially higher in female than in male whiteflies (Fig. 2B). The higher densities may be related to physiological differences such as the exclusive female role in the vertical propagation of symbionts (i.e., transovarial transmission). One hypothesis is that a high *Hamiltonella* density in females may support an increased production of nutritional and other metabolites required for fertility and may also result in greater transmission of symbionts to progeny. Alternatively, our recent work suggested that *Hamiltonella* confers fitness benefits and acts as a nutritional mutualist [51], [52]. The benefits provided to whiteflies by *Hamiltonella* infection could explain the high frequency of *Hamiltonella* in *B. tabaci* B and Q in China [26], [27].

### Response of Symbionts to Virus Infection

*Portiera* and *Hamiltonella* densities were unaffected by the virus (TYLCV) status of the whitefly hosts (Fig. 4A and 4B). These findings suggest that the localization of these symbionts in bacteriocytes (Fig. 1B) may limit their response to plant virus or that the bacteria are resistant to viral effects as a result of the long co-evolutionary history of the bacteria and whiteflies [29]. In contrast to *Portiera* and *Hamiltonella* densities, *Rickettsia* densities were higher in viruliferous than in non-viruliferous females of *B. tabaci* B based on both copy number (Fig. 4C and 4D) and fluorescent *in-situ* hybridization (FISH) (Fig. 5A–C). The location of *Rickettsia* outside of bacteriocytes (Fig. 1A) may explain why *Rickettsia* desity was weakly regulated and enhanced by the presence of TYLCV in *B. tabaci* B females. The strong response of *Rickettsia* to TYLCV infection might result from the bacterium sensing of signals released by the virus or by the viruliferous host. It remains to be determined whether the regulation of *Rickettsia* density in males is host- or symbiont-mediated. Perhaps the sex-specific response of *Rickettsia* to TYLCV is consistent with the prediction that benefits more from an increase in its density in female than in male whiteflies, as *Rickettsia* infection in whiteflies had reproductive advantage along with the substantial performance benefits for females to facilitate the spread of *Rickettsia* [35].

Nearly 80% of viral infection of plants is due to insect transmission [20], and interaction between symbiotic bacteria and viruses in insects are common. Recently, TYLCV was shown to affect or suppress the immune response of the host whitefly [53], and this may allow *Rickettsia* increased opportunity to multiply in the host. The variation in *Rickettsia* density in our study suggests that the host whitefly may have poor control of *Rickettsia* because of the symbiont’s location outside of bacteriocytes. *Rickettsia* has been shown to influence several aspects to the whitefly biology [34]–[36] and may activate the expression of stress and immunity-related genes to prime the whitefly for stress conditions, as shown for whitefly resistance to heat [34]. Other symbiotic bacterium such as *Wolbachia* increases resistance to RNA viruses has been shown in dipterans [54], [55] including *Culex quinquefasciatus* and *Aedes aegypti* [56], [57]. The enhanced resistance mediated by *Wolbachia* might be regulated by tolerance of virus infection, accumulation, or a combination of both mechanisms and from the host’s innate immune system being primed by both *Wolbachia* and a virus [56]. The response of *Rickettsia* to TYLCV infection may affect whitefly–TYLCV interactions, and the immune response gene in *B. tabaci* induced by *Rickettsia* needs to be further studied.

### Response of Symbionts to Temperature

*Portiera* and *Hamiltonella* densities were not affected by temperature treatment (Fig. 6A and 6B), which suggests that their response to temperature is limited by their localization in bacteriocytes. The density of *Rickettsia*, which was not located in bacteriocytes in our *B. tabaci* B population, was significantly reduced at 15°C and was either moderately reduced (in the first generation) or not affected (in the second and third generations) at 35°C (Fig. 6C). These different effects of low and high temperature may provide clues as to how the symbiosis is controlled. A similar pattern was observed for *Spiroplasma* in drosophilid flies exposed to high and low temperatures [58]. Previous studies have demonstrated that, in various insect-symbiotic systems, symbiont density was affected by symbiont genotype, symbiont localization pattern, host genotype, and environmental factors [37], [59]–[61]. The different dynamics of *Rickettsia* vs. *Portiera* or *Hamiltonella* in our study may be attributable to symbiont location, i.e., *Rickettsia* responds more strongly than *Portiera* or *Hamiltonella* to temperature (and probably to other environmental effects) because unlike *Portiera* or *Hamiltonella*, *Rickettsia* is located outside of the bacteriocytes. Our results are also in accord with Brumin et al. (2011) [34], who reported that heat tolerance was higher for whiteflies containing *Rickettsia* located outside bacteriocytes rather than in bacteriocytes and that this heat tolerance was associated with a reduction in *Rickettsia* numbers. Because they are located in bacteriocytes, *Portiera* and *Hamiltonella* might gain some protection from environmental change, and this might help them to obtain or synthesize nutrients [1], [52]. To date, the role of *Rickettsia* located outside bacteriocytes in *B. tabaci* nutrition has not been investigated. Our results demonstrated that high temperature reduced the transmission and maintenance of *Rickettsia* in the first generation but not in subsequent generations. The mechanism by which the subsequent generation of *Rickettsia* became tolerant of high temperature is unknown. Researchers have suggested that the presence of *Rickettsia* outside bacteriocytes induced host cytoskeleton genes that indirectly contributed to thermotolerance of *B. tabaci* B [34]. This interaction suggested that cytoskeleton proteins were potential targets of protective mechanisms. One hypothesis that may explain our results is that the reduced *Rickettsia* amounts induce the expression of cytoskeleton genes. This specific group of genes may indirectly contribute to heat tolerance and regulate the restoration of *Rickettsia* density. With regard to temperature, *B. tabaci* is one of the most devastating agricultural pests in tropical and sub-tropical countries [42], and the high temperature of condition 35°C examined in this study is within the range of natural conditions for *B. tabaci*. Hence, it appears plausible that higher temperature has limited effects on the symbionts densities in the whitefly. In natural *B. tabaci* B populations in China, infection frequencies of *Rickettsia* are generally high [26], [27]. The high temperature-dependent stability of vertical transmission may be relevant to the high infection frequencies of *Rickettsia* observed in natural *B. tabaci* B populations. In this study, constant temperature treatments were used. Additional insight into symbiont infection dynamics in nature could be gained by experiments that used fluctuating temperature regimes.

## Conclusion

In summary, our experimental results provide the first evidence that the densities of *Rickettsia* located outside bacteriocytes respond strongly to virus infection and low temperature. These results suggest that the regulation of symbiont density results from co-adaptive processes. The coordination of density may reduce conflict between symbiotic partners and thereby drive inter-specific associations and promote specialization of established symbioses.
